# Bone metastasis from noninvasive follicular thyroid neoplasm with papillary-like nuclear features (NIFTP); a case report

**DOI:** 10.1186/s12902-021-00883-7

**Published:** 2021-11-04

**Authors:** Yasaman Fakhar, Alireza Khooei, Atena Aghaee, Hadis Mohammadzadeh Kosari, Leonard Wartofsky, Seyed Rasoul Zakavi

**Affiliations:** 1 Nuclear Medicine Research Center, Mashhad University of Medical Sciences, Mashhad, Iran; 2 Pathology Department, Ghaem hospital, Mashhad University of Medical Sciences, Mashhad, Iran; 3 Thyroid Cancer Research Unit, MedStar Health Research Institute, Georgetown University School of Medicine, Washington, DC, USA

**Keywords:** NIFTP, Bone metastasis, Thyroid cancer, Thyroglobulin, Case report

## Abstract

**Background:**

The term non-invasive follicular thyroid neoplasm with papillary-like nuclear features (NIFTP) was recently proposed as a non-malignant thyroid lesion with indolent behavior that does not require post-operative radio-iodine treatment. We are reporting a case of NIFTP with bone metastasis that is the second case reported so far.

**Case presentation:**

We describe a 38-year-old woman who presented with an indeterminate thyroid nodule and underwent total thyroidectomy with the finding of NIFTP on careful pathologic examination. However, her initial follow-up evaluation revealed a serum thyroglobulin level of > 300 ng/ml and a diagnostic whole body ^131^I scan demonstrated a focus of increased uptake in the left hemipelvis, confirmed on CT scan to be a lytic lesion in the left iliac bone. She was treated with 7.4GBq (200 mCi) of ^131^I and her follow-up 1 year later revealed an undetectable serum thyroglobulin and a negative whole body ^131^I scan with no visible uptake in the iliac bone indicating an excellent response.

**Conclusion:**

This case presentation reminds us to be alert to the rare occurrence of distant metastasis in NIFTP and the need for a case by case analysis and continuing post-operative follow-up for detection of residual or recurrent disease.

## Background

The term non-invasive follicular thyroid neoplasm with papillary-like nuclear features (NIFTP) was recently proposed to describe a non-malignant lesion with indolent behavior. Given an anticipated benign outcome, conservative management by either lobectomy or thyroidectomy has been recommended without necessity for either post-operative radio-iodine ablation or suppressive levothyroxine therapy [1]. The more specific delineation of this neoplasm was introduced to stratify the low-risk group of thyroid cancers more precisely [2]. Confirmation of NIFTP and exclusion of a potentially invasive thyroid neoplasm can only be made by an experienced pathologist based on strict criteria of inclusion and exclusion [3]. As described, the NIFTP designation was based upon pathologic analysis of 109 patients with noninvasive encapsulated follicular variant of papillary thyroid carcinoma (EFVPTC) who had demonstrated no evidence of disease after 10-26 years of follow up [1]. As we had noted earlier, cautious acceptance of the certainty of benign outcomes with these tumors is warranted by the retrospective nature of the latter report, and the fact that it was based upon a relatively small number of patients in 13 sites in 5 different countries without validation [4]. That caution is justified by reports of the presence of lymph node metastases in patients with EFVPTC [5–7] as well as one case of bone metastasis [5]. Our case is the second reported bone metastasis in patients with NIFTP and shows the importance of complete evaluation of these patients including thyroglobulin measurement in initial evaluation. We recommend post-operative surveillance of patients with NIFTP with neck examination and serial measurements of thyroglobulin for 2-3 years to confirm a benign outcome. Such evaluation allowed early detection of bone metastasis in our patient with NIFTP and an opportunity for definitive therapy.

## Case presentation

**Fig. 1:**
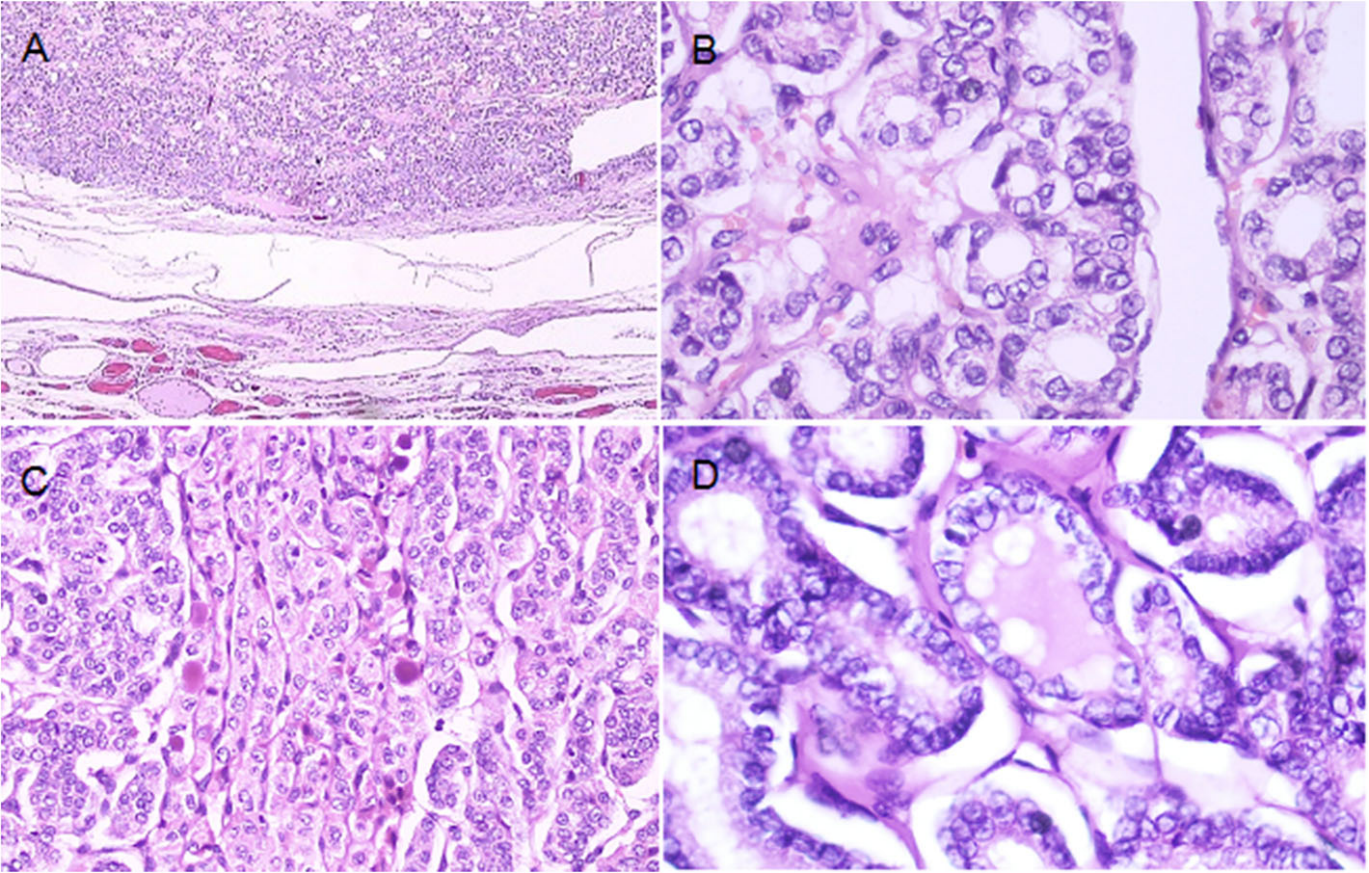
The tumor is entirely encapsulated with a sharp margin, H/E stain (40X) (A). PTC-like nuclear features are seen more frequently in microfollicular (B) and trabecular foci (C) of the tumor, H/E stain (200X). Nuclear overcrowding, ground glass appearance, nuclear groove and less frequently nuclear inclusion are seen, H/E stain (400X)(D)

**Fig. 2:**
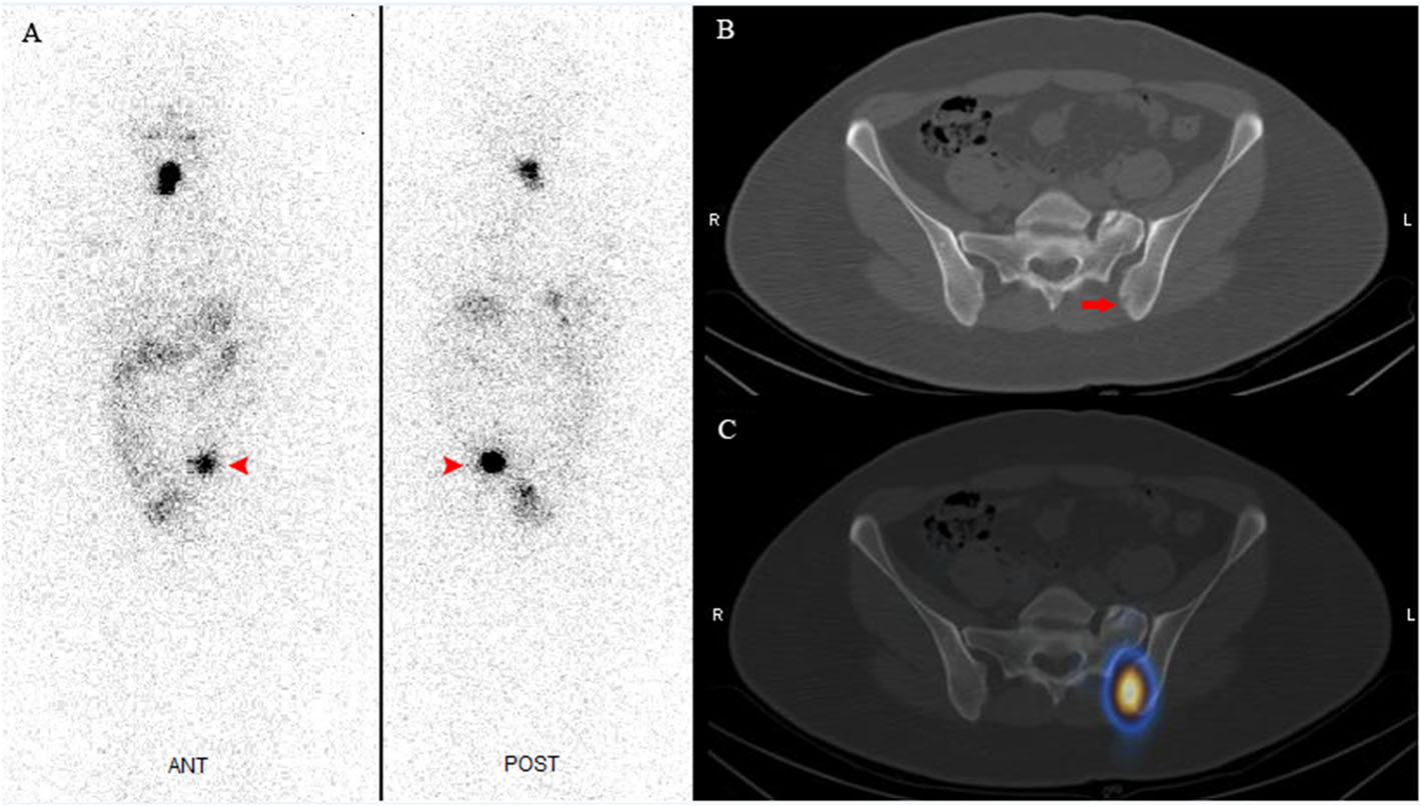
Diagnostic whole body iodine scan shows post-surgical thyroid remnant (PSTR) and a focus of abnormal uptake in the left hemipelvis (A; *arrowhead*). SPECT/CT from the pelvic region shows a lytic lesion in the left iliac bone (B; *arrow*), with intense iodine 131 uptake (C)

The present case describes a 38-year-old woman with history of multinodular goiter who underwent total thyroidectomy prompted by the presence of a suspicious dominant nodule. She had no history of other disease and had no complaints other than mild and dull pain in the right side of pelvis. Careful histopathologic examination of the thyroid mass revealed an intact capsule and no evidence of vascular or capsular invasion or extranodular tumor, but PTC-like nuclear features including nuclear overcrowding, ground glass appearance and nuclear grooves were present (Fig. 1), prompting a diagnosis of noninvasive follicular thyroid neoplasm with papillary-like nuclear features (NIFTP). One month after thyroid surgery, and no thyroid hormone administration, serum thyrotropin (TSH) was elevated to 37.3μIU/ml with a concomitant serum thyroglobulin (Tg) level of > 300 ng/ml and anti-Tg Ab of 17 IU/ml. Cervical examination and ultrasonography did not reveal any evidence of lymphadenopathy that might indicate lymph node metastasis. A diagnostic whole body iodine scan (WBIS) was performed 48 h after administration of 37 MBq (1 mCi) of I-131 and showed a small post-surgical thyroid remnant (PSTR) and a focus of abnormal intense uptake in the left hemipelvis. A SPECT/CT examination of the pelvis demonstrated a lytic lesion in the left iliac bone corresponding to the focus of iodine 131 uptake (Fig. 2).

**Fig. 3:**
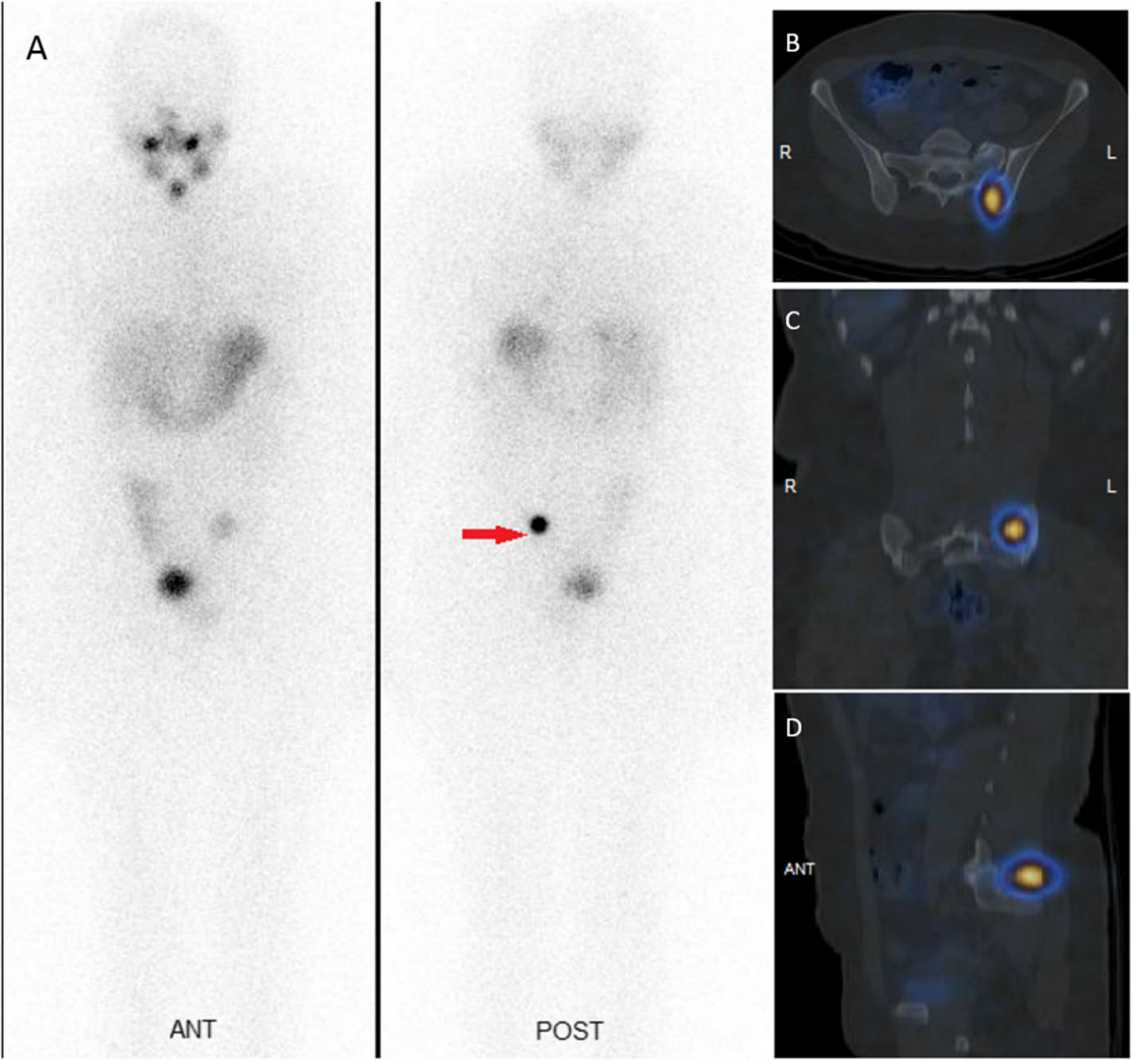
Post ablation whole body iodine scan (A) demonstrates increased focus of iodine uptake in the left hemipelvis and SPECT/CT of the pelvic region (B-D) localized the activity to the left iliac bone

Although the presence of radioiodine uptake in the iliac bone lesion clearly suggested a thyroid origin, a full metastatic work-up including abdominopelvic ultrasonography was performed and was negative, ruling out an alternative neoplasm. The patient was treated with 7.4GBq (200 mCi) of I-131 and the post ablation WBIS and SPECT/CT of the pelvic region performed 5 days later (Fig. 3) again confirmed an increased focus of iodine uptake in the left iliac bone with associated lytic change on the CT component.

**Fig. 4:**
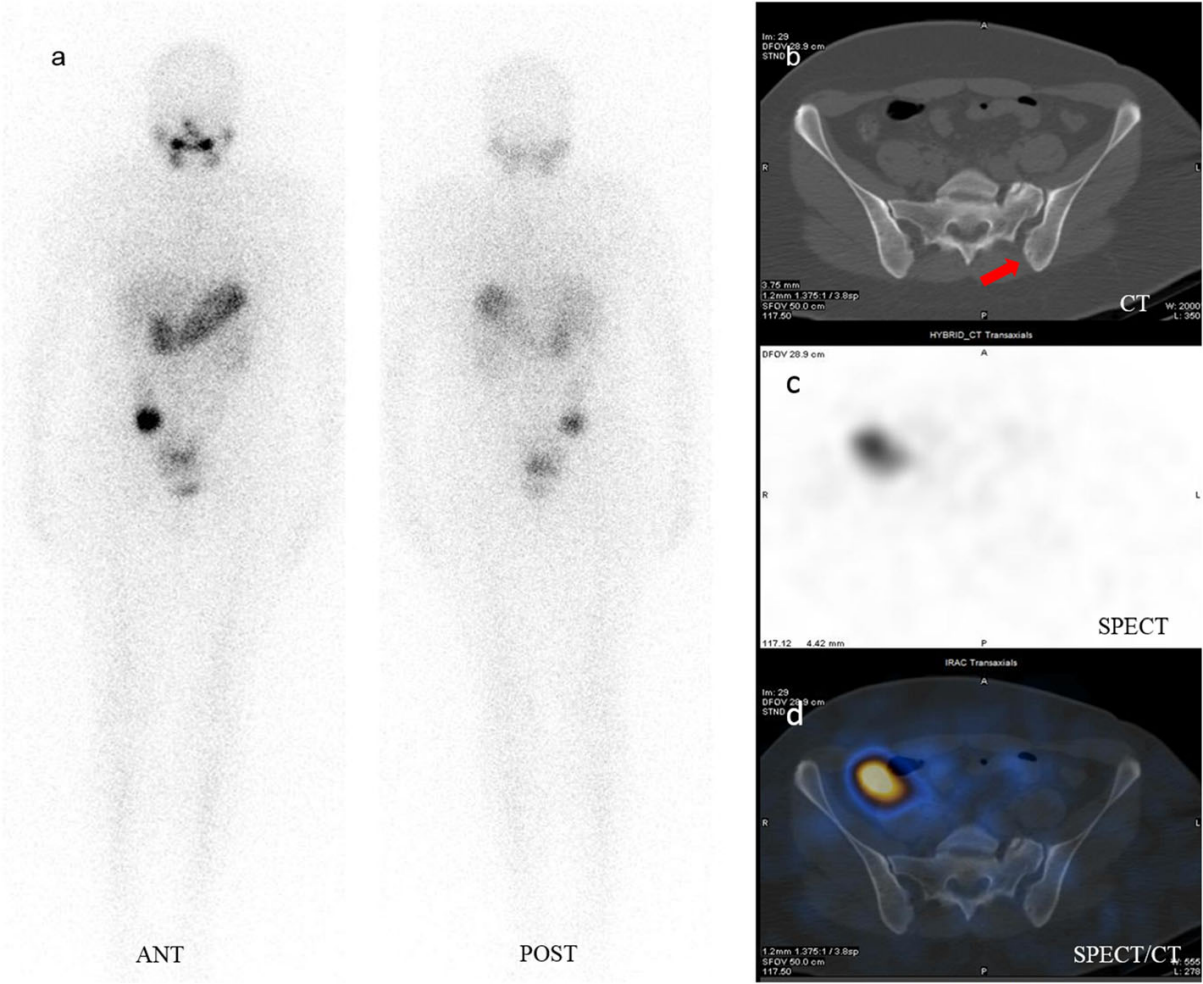
Follow-up whole body iodine scan one year after treatment, showed normal distribution of radioiodine activity throughout the body (a). On pelvic CT, the small lytic lesion again was noted in the left iliac bone (b), but with no corresponding iodine uptake on SPECT and SPECT/CT views (c, d)

Following radioiodine therapy, she received thyroid hormone therapy for TSH suppression and underwent continued surveillance with measurements of Tg and anti-Tg antibody levels. One year post-radioiodine therapy serum Tg was decreased to < 0.2 ng/ml and a WBIS performed after thyroid hormone discontinuation, revealed no iodine activity throughout the body. SPECT/CT images from the pelvis showed the prior small lytic lesion in the left iliac bone but with no corresponding radioiodine uptake (Fig. 4). She has no compliant and was happy with the treatment.

### Discussion and conclusions

NIFTP is now considered a non-malignant indolent lesion and a rigorous histological examination is critically important for its diagnosis. Criteria for diagnosis include encapsulation, a purely follicular pattern and the presence of nuclear features of papillary thyroid cancer, while lacking capsular and vascular invasion, high-grade features, and a significant component of solid growth [1, 3].

In a recent review of 265 NIFTP patients, no patient with lymph node metastasis or recurrence was found and only one patient with pulmonary metastases was reported [8]. In addition to the cytological examination, cervical ultrasonography and molecular tests can be useful for diagnosis of suspicious cases of NIFTP [9, 10]. Clinicians and pathologists should be familiar with both the ultrasonographic features of these tumors as well as the diagnostic pitfalls inherent in cytologic and histologic diagnosis of NIFTP [8]. Because diagnosis of NIFTP may be made less frequently, e.g. in Asian populations, and considerable observer variation in diagnosis of NIFTP has been reported [11], strict histologic criteria must be applied for diagnosis as was done in the present case.

Bone metastasis of thyroid cancer is generally associated with reduced overall survival and is best managed with a multidisciplinary approach [12].

While patients with metastases to bone may demonstrate radioiodine uptake, the lesions generally are either less radiosensitive or fail to collect sufficient radioiodine for cure. Moreover, a sizable fraction of bone metastases do not trap radioiodine at all and are completely refractory to I-131 treatment [13]. Nevertheless, patients with bone metastases treated with I-131 and who achieve complete radioiodine response will show significantly higher 15-year survival than those not treated [14]. The bone metastasis in our patient showed both positive radioiodine uptake on scan and a salutary response to a therapeutic dose of I-131 based upon the fall in levels of serum Tg to undetectable and the subsequent negative scan 1 year later. Multiple factors have been reported to be associated with favorable response to radio iodine therapy in cases of bone metastasis and include: younger age, high levels of 131I uptake in the metastases, solitary osseous metastasis, performing surgery before RAI therapy, and the time frame of RAI administration [14]. In the present case, both the high uptake of radio-iodine in the solitary bone metastasis and the relative youth of the patient were good prognostic factors.

The case presented illustrates that although the overwhelming majority of patients with NIFTP will have an excellent prognosis and outcome [8], physicians should be alert to the rare but potential occurrence of metastatic disease and the need for case by case analysis and continuing post-operative surveillance for detection of residual or recurrent disease, at least during the initial evaluations. As measurement of serum Tg is a well-established marker for residual or recurrent thyroid cancer [15], we suggest measurement of serum Tg and anti-Tg antibody levels to establish a post-operative baseline concentration and then serial estimates of Tg every 6 months for the first 2 years after initial treatment. This recommendation is consistent with that of Baloch et al. who advised that continuing prospective follow up of these patients was necessary to verify that their post-operative course would be truly indolent [16].

## Data Availability

All data generated or analysed during this study are included in this published article [and its supplementary information files].

## Abbreviations

NIFTP: Non-invasive follicular thyroid neoplasm with papillary-like nuclear features
CT: Computed Tomography
EFVPTC: Encapsulated Follicular Variant of Papillary Thyroid Carcinoma
TSH: Thyroid Stimulating Hormone
PTC: Papillary Thyroid Carcinoma
Tg: Thyroglobulin
Anti-Tg Ab: Anti Thyroglobulin Antibody
WBIS: Whole body iodine scan
PSTR: Post-surgical thyroid remnant
SPECT: Single photon emission tomography
RAI: Radio-active iodine
